# Comparison between Deflection and Vibration Characteristics of Rectangular and Trapezoidal profile Microcantilevers

**DOI:** 10.3390/s90402706

**Published:** 2009-04-16

**Authors:** Mohd. Zahid Ansari, Chongdu Cho, Jooyong Kim, Booun Bang

**Affiliations:** Department of Mechanical Engineering, Inha University, 253 Yonghyun-dong, Nam-Ku, Incheon, 402-751 Republic of Korea

**Keywords:** Sensor, Surface stress, Microcantilever, Resonant Frequency, Deflection

## Abstract

Arrays of microcantilevers are increasingly being used as physical, biological, and chemical sensors in various applications. To improve the sensitivity of microcantilever sensors, this study analyses and compares the deflection and vibration characteristics of rectangular and trapezoidal profile microcantilevers. Three models of each profile are investigated. The cantilevers are analyzed for maximum deflection, fundamental resonant frequency and maximum stress. The surface stress is modelled as in-plane tensile force applied on the top edge of the microcantilevers. A commercial finite element analysis software ANSYS is used to analyze the designs. Results show paddled trapezoidal profile microcantilevers have better sensitivity.

## 1. Introduction

Although generally used in topological investigations of surfaces such as in atomic force microscopy, arrays of microcantilevers are attracting much interest as sensors in a variety of applications. Microcantilever sensors have emerged as a very powerful and highly sensitive tool to study various physical, chemical, and biological phenomena. The physical phenomena can be calorimetric [1], rheometric [2], optical switching [3], acoustic [4], infrared [5], surface stress and magnetoelastic stress [6], and so on. As chemical sensors, microcantilevers have been used as pH meters [7], NO_2_ sensors [8], atrazine pesticide detectors [9], etc. However, it is the biosensing applications that are attracting the most interest in microcantilevers. Owing to their label-free, rapid and real-time detection abilities, arrays of microcantilevers are becoming increasingly popular in biosensing applications. As biosensors, microcantilevers have been used in applications such as DNA hybridization [10], biomarking of myoglobin and kinase proteins [11], detection of biomarker transcripts in human RNA [12], assaying amyloid growth and protein aggregation [13], and DNA hybridization using hydration induced tension in nucleic acid films [14].

Surface stresses, in general, are generated either by the redistribution of the electronic charge at the surface due to the change in the equilibrium positions of the atoms near the surface, or by the adsorbtion of foreign atoms onto its surface to saturate the dangling bonds [15]. Microcantilever biosensors exploit the adsorbate-induced surface stress change in measuring and assaying the unknown species present in a media. When the analyte molecules are put onto the functionalized cantilever surface, a biomolecular reaction takes place and the analyte molecules are adsorbed onto the cantilever surface. The adsorption alters the surface stress distribution on the adsorbing surface and results in cantilever motion. Since the induced surface stress strongly depends on the molecular species and its concentration, by measuring the cantilever deflection the attaching species as well as its concentration can be determined.

Microcantilever biosensors commonly use optical lever readout technique to observe the deflection. In practice, the accuracy in the deflection measurements not only depends on the actual deflection occurred but also on the signal-to-noise ratio. Most of the noise in the signal can be attributed to the thermal drift. To improve the signal-to-noise ratio, the resonant frequency of the cantilever should be made as large as possible. Thus, to increase the overall cantilever sensitivity, we should select a design that shows both higher deflection and higher resonant frequency. The sensitivity of a cantilever can be changed by changing the cantilever material, shape, size, or profile. Polymeric materials such as polyethylene terephthalate (PET) [16] and SU-8 have been used as alternate cantilever materials [17–19]. The main advantage in using polymeric microcantilevers lies in their low elastic modulus, which greatly improves the cantilever deflection. In addition, polymeric microcantilevers are easy and inexpensive to fabricate. However, polymer cantilevers are highly temperature sensitive and require fine control of the surrounding [18–20]. By changing the shape of rectangular profile microcantilever, Ansari and Cho [21] proposed a new design that shows an increase of 75% in the deflection produced in a rectangular microcantilever. They also proposed a deflection contour relating the deflection and the cantilever size for a given surface stress. Villanueva *et al.* [22] successfully used U-shaped piezoresistive cantilevers for measuring biomolecular forces of the order of 65 pN. Fernando *et al*. [23] carried detailed analysis on relation between deflection and resonant frequencies for various cantilever profiles.

To increase simultaneously the deflection and resonant frequency of a microcantilever, this paper investigates the deflection and vibration characteristics of rectangular and trapezoidal profile microcantilevers having three different shapes. These cantilevers can be used as the sensing element in biosensors. First, we separately analyze the effect of cantilever profile change and the effect of cantilever shape change, and then combine the profile change with the shape change to investigate the deflection and resonant frequency of the microcantilevers. All the cantilevers were investigated for maximum deflection occurred, fundamental resonant frequency, and maximum induced stresses. The surface-stress induced deflection in the microcantilever is modelled by an equivalent in-plane tensile force acting on the top edge of the cantilever, in the length direction. A commercial finite element method (FEM) software ANSYS is used in this analysis.

## 2. Theory

Microcantilever biosensors exploit surface-stress induced deflections to assay the target molecules. When the target molecules attach onto the functionalized top surface of the cantilever, the surface stress distribution on this surface is changed, resulting in a differential stress across the top and bottom surfaces of the cantilever. The differential stress ultimately generates deflections in the cantilever. For a rectangular profile microcantilever, the differential surface stress (Δ*σ*) and deflection (Δ*z*) are related by Stony Equation given as [24]:

(1) $$\Delta z=\;\;\frac{3(1-\nu )\;\Delta \sigma }{E}{\left(\frac{l}{t}\right)}^{2}$$

where *l* and *t* are the length and the thickness of the cantilever, and *E* and *ν* are the elastic modulus and Poisson ratio of the cantilever material. The Stoney Equation is however not very accurate in predicting deflections, mainly due to the violation of no-constraints condition used to derive it. Using finite element analysis, Dahmen *et al.* [25, 26] showed that constraining and anisotropy can induce measurement errors as high as 50% in determining the surface stress or the magnetoelastic constant. A similar observation was made by Sader [27]. Therefore, in this study we used a form of Stoney Equation used in [12]:

(2) $$\Delta z=\;\;\frac{4(1-\nu )\;\Delta \sigma }{E}{\left(\frac{l}{t}\right)}^{2}$$

The fundamental resonant frequency (*f*_0_) for a rectangular profile cantilever of mass density (*ρ*) is given as:

(3) $$f_{0}=\;\;\frac{1}{2\pi }\sqrt{\frac{E}{\rho }}\cdot \frac{t}{l^{2}}$$

As can be seen from Equations 2 and 3, any attempt to increase the sensitivity by increasing the deflection will decrease the resonant frequency. In fact, the two equations indicate an inverse relation between them. For instance, following Equation 2, if we try to increase the deflection by increasing the length or decreasing the thickness, Equation 3 predicts an opposite effect for the frequency. Thus, the deflection and frequency are coupled terms; and hence, should be treated in such manner. Combining Equations 2 and 3, we define sensitivity (Δ*z ·f*_0_) as:

(4) $$\Delta z\cdot f_{0}=\;\;\frac{2(1-\nu )\;\Delta \sigma }{\pi \sqrt{E\rho }}\cdot \frac{1}{t}$$

Thus, instead of increasing deflection or resonant frequency individually, it is more practical to increase the overall sensitivity predicted by Equation 4. In other words, comparing sensitivities is a better way to compare the suitability of a microcantilever design. Therefore, in this study we also calculated and compared the sensitivity values of all the cantilever models. To select the best cantilever model, we should choose one that has higher Δ*z ·f*_0_ value, with more inclined towards the deflection.

For a microcantilever of trapezoidal profile, *t* (*x*) = *t*_l_ + (*t*_0_ – *t*_l_) *x*/*l*, the Stoney Equation (Equation. 2) can be given as [23]:

$$\Delta z=\;\;\frac{8\;(1-\nu )\;\Delta \sigma l^{2}}{E{\left(t_{0}-t_{l}\right)}^{2}}\left[\text{ln}\left(\frac{t_{0}}{t_{l}}\right)+\frac{t_{l}}{t_{0}}-1\right]$$

where *t*_0_ and *t*_l_ are the thicknesses of the cantilever at the fixed and free ends. This study used *t*_l_ = *t*_0_/2. Hoffman and Wertheimer [28] gave a simple and accurate formula for calculating the fundamental resonant frequency for a beam of trapezoidal profile:

(5) $$f_{0}=\;\;C\sqrt{\frac{S}{M}},\text{where}\;C=\;\;c_{1}\sqrt{\frac{1}{c_{2}c_{3}}}$$

In this equation, *S* is the spring constant, *M* is cantilever mass, and *c*_1_, *c*_2_ and *c*_3_ are tapering-ratio dependent mass distribution parameters.

## 3. Modelling and Simulation

**Figure 1.**
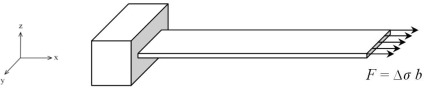
Modelling the surface stress induced deflection in a microcantilever by in-plane tensile force acting on the top surface.

The surface-stress induced deflection in a microcantilever can be modelled by applying a lengthwise in-plane tensile force at the free edge of the top surface of the cantilever (Figure 1). Since surface stress is expressed in unit of force per unit width, multiplying the surface stress by the cantilever width will give the total tensile force acting on the top surface. To verify this model, we will use it to simulate the experimental result reported in [11]. Using an array of eight silicon rectangular microcantilevers Arntz *et al*. [11] reported that a maximum surface stress of 0.05 N/m is generated upon injection of 50 μg ml^−1^(∼2.5 μM) myoglobin protein onto the functionalized surface of the microcantilever. The surface stress resulted in a maximum deflection of 0.89 μm at the cantilever free end. The cantilever size was 500×100×0.5 μm, and the elastic modulus and Poisson ratio was 130 GPA and 0.28, respectively. This cantilever is used as a reference in this analysis.

**Figure 2.**
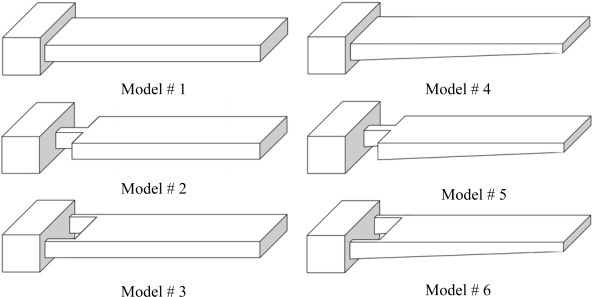
Schematic designs for the rectangular and trapezoidal profile cantilevers. All the models have same length and fixed-end thickness.

Figure 2 presents the rectangular and trapezoidal profile cantilevers analyzed in this study. As can be seen in the figure, three different shapes for each profile are analyzed. One cantilever of each type has uniform width throughout its entire length (Models #1 and #4). The remaining two have non-uniform width with their widths reduced towards the fixed end (Models #2, #3 and #5, #6). In these models, one or two rectangular holes are introduced at their fixed ends. Models #2 and #5 have two rectangular holes of size 50×40 μm each on either side of their fixed ends. In contrast, Models #3 and #6 have only one rectangular hole of size 50×80 μm in the middle of their fixed ends. All the designs are 500 μm long, 100 μm wide at their free ends and 1 μm thick at their fixed ends. For trapezoidal profile cantilevers, the free end thickness is half the fixed end thickness, i.e. *t*_l_ = *t*_0_/2. For simulations, a FEM software ANSYS Multiphysics was used to calculate the deflection, fundamental resonant frequency and maximum stress induced. The simulations were performed on three-dimensional FE models of the cantilevers, under linear, static conditions. The FE models were meshed by SOLSH190 elements. As shown in Figure 1, a tensile force of *F* = 0.05 N/m×100×10^−6^ m = 5×10^−6^ N/m was applied to the top free edge of all the six models.

## 4. Results

To ascertain the validity of modelling surface-stress induced deflection by in-plane tensile force, Table 1 compares the experimental, analytical and simulation results for a given surface stress. All the simulation parameters in this analysis were adopted from reference [11].

In Table 1, the experimental results are from [11], the analytical results are obtained using Stoney Equation (Equation 2) and simulation results are from FEM using ANSYS. A comparison among the three results shows the analytical and simulation results have good accord indicating the accuracy of Equation 2. However, as can be seen in the table, the experimental result deviates significantly from the analytical and simulation results. The reasons behind this deviation are explained in the ‘Discussion’ section.

Table 2 shows a comparison between the analytical and simulation results for maximum deflection and resonant frequency of uniform width rectangular and trapezoidal profile cantilevers. For calculating the deflection and frequency values for the rectangular cantilever (Model #1), Equations 2 and 3 were used. Similarly, for uniform width trapezoidal cantilever (Model #4), Equations 4 and 5 were used. As can be observed in Table 2, the analytical and simulation values for both cantilever types show very similar results, indicating the conformity of the analysis.

**Figure 3.**
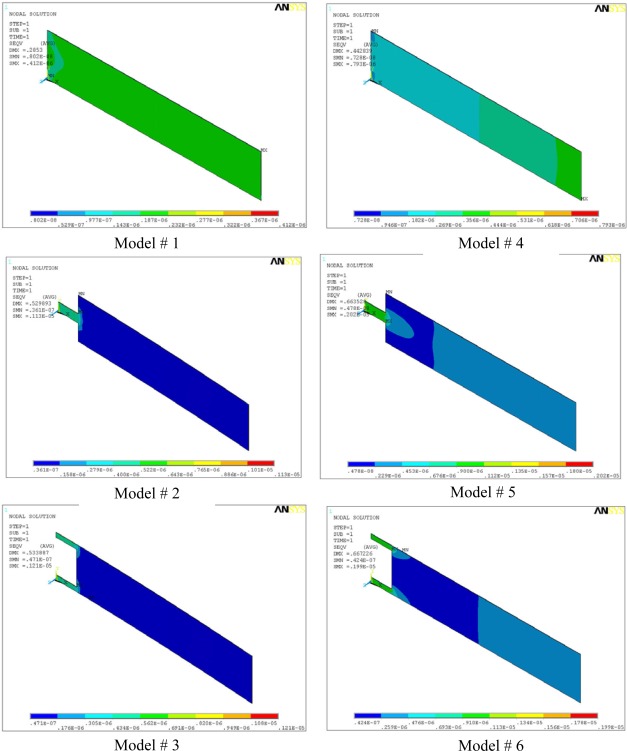
Von Mises stress distribution in the microcantilever models. Models #1, #2 and #3 have rectangular profiles, and Models #4, #5 and #6 have trapezoidal profiles.

Figure 3 shows the stress distribution in all the microcantilever models. The maximum stress (SMX) and the maximum deflection (DMX) values are also indicated in the top-left corner of the micrographs. In the analysis, we used micrometre as unit of length and newton as unit of force. Accordingly, in the figure, the cantilever size and deflections are expressed in micrometers, and the stresses are in TPa (i.e., 10^6^ MPa). The maximum induced stresses range from a minimum of 0.41 MPa for Model #1 to a maximum of about 2 MPa for Models #5 and #6. A stress comparison between Models #1 and #4 shows that profile change alone increased the stresses from 0.41 MPa to 0.79 MPa. The deflection and stress values for Models #2 and #3 are almost equal. Similar observation is true for #5 and #6. This behaviour is expected because from mechanics of material point of view, Models #2 and #3 are identical because they have same flexural stiffness, i.e. their resistance to bending is equal. Same observation holds for #5 and #6. However, it should be noted that Models #3 and #6 have better torsion resistance than #2 and #5, and therefore should be preferred.

The changes in the cantilever profile or the cantilever shape will lead to a change in the area which will introduce sharp corners in the cantilever. The sharp corners in the microcantilever models can raise the stress concentration factors by many folds. As we can see in the Figure 3, the maximum stress in Model #6 is about six times the Model #1, and the maximum stresses are located at the corners near the fixed end of the models. Sooriakumar *et al*. [29] reported that the sharp corners introduced by anisotropic etching reduce the ultimate strength of silicon to about 300 MPa. Therefore, an ultimate strength of 300 MPa is more practical for analyzing the design strength of silicon microcantilevers. The maximum induced stress of 2.02 MPa, in Model #5, is still much lower than 300 MPa for silicon; therefore, we can conclude that all the models are safe and will not fail under normal conditions.

Table 3 compares the simulation results for maximum deflection (Δ*z*), fundamental resonant frequency (*f*_0_), sensitivity (Δ*z · f*_0_), and maximum stress induced (*σ*_max_) for all the six models shown in Figure 2. The corresponding values for the reference cantilever are also listed. The maximum stress induced in all the models lies between one and five times. The change is stress values in the models is understandable because any change in the shape or profile of the cantilevers will result in a change in area, leading to change in the stress values.

From Table 3 it is obvious that deflections can be increased just by changing the cantilever shape. For instance, comparing the deflections indicated by Model #1 with Models #2 and #3, we can easily observe an increase of 89% in deflections induced in both cases. Similarly, for trapezoidal profile cantilever, changing the cantilever shape from Model #4 to either Model #5 and #6 increases the deflection by about 51%. However, the shape change in both cantilever types decreases their resonant frequency as well.

Another approach to simultaneously increase the deflection and frequency is to change the cantilever profile. Comparing the deflection shown by Model #1 to Model #4, we observe about 57% increase in deflection and about 9% increase in the resonant frequency. Furthermore, it can be easily observed in Table 3 that among the models, the reference model has highest deflection as well as Δ*z · f*_0_ value, making it most suitable cantilever. However, since this cantilever also has the least value of the resonant frequencies, it will have the smallest signal-to-noise ratio. Therefore, the reference cantilever is unsuitable under dynamic conditions. Except for Model #4, all the models suggest any increase in deflection is accompanied with a decrease in frequency. Thus, Model #4 seems most suitable to be used as sensing element in microcantilever biosensor. However, if dynamic properties of the cantilever are not a major concern, Models #5 and #6 show highest deflection and sensitivity values, and are more appropriate to increase the overall sensitivity of the biosensor.

## 5. Discussion

As mentioned earlier, Table 1 shows a significant difference between the experimental and the analytical and simulation results for deflection. The analytical value was calculated using the Stoney Equation (Equation 2), and the simulation value was determined using in-plane force model. The deviation in the deflection results can be explained by the linear and nonlinear relations between the deflection and the surface stress. Linear relation implies a linear relation between the load applied and the deflection produced. As can be seen in Equation 2, the deflection is linearly proportional to the surface stress; therefore, Equation 2 is a linear equation. Since the length-to-width ratio of the experimental cantilever is high (i.e., *l*/*b* = 5), general plate theory can be applied to it. In addition, since the width-to-thickness ratio of the cantilever is also very high (i.e., *b*/*t* = 1,000), the cantilever can be safely classified as a thin plate. In general, a plate bending problem becomes nonlinear when the deflection values exceeds one-half the plate thickness, because the deflection then becomes nonlinear and produces stiffening effect in the plate [30]. The higher the deflection higher will be the nonlinear effect. The stiffening effect in the plate increases its bending stiffness, resulting in reduced deflection in the plate.

In the experimental case, the cantilever thickness is 0.5 μm and the deflections predicted by analytical and simulation is about 1.1 μm (Table 1). Since the experimental deflection is more than two times the cantilever thickness, or about five times the one-half plate thickness, experimental case is clearly a large deflection problem. And, stiffening will occur in the cantilever, leading to a reduction in the deflection. Thus, for large deflection and nonlinear cases deflections predicted by the Stoney Equation will be lower than the experimental values. This observation is evident in Table 1. Compared with the experimental deflection result of 0.89 μm, the analytical and simulation results show a higher value of 1.11 and 1.14 μm, respectively. Thus, we may conclude that the reduced deflection observed in the experiment can be attributed to the stiffening effect in the cantilever. The deflection value using nonlinear analysis is 0.93 μm [21], which is very close to the experimental result. Since for all the six models investigated, the deflections predicted by the Stoney Equation are comparable to the cantilever thickness, linear simulation conditions can be applied to our analysis. Therefore, in this study, all the simulations were done under linear conditions.

The dynamic properties of microcantilevers used in biosensors are critical in accurate measurement of deflections. In practical applications, there can by thermal, structural, or flow induced excitations that can interfere with and hence produce noise in the signals. Therefore, it is vital to eliminate or isolate the noise in the signal, and to insure that the deflections induced are solely due the surface stress change. To prevent noise, a cantilever should have high signal-to-noise ratio, which can be achieved by making the resonant frequency of the cantilever as large as possible. The fundamental resonant frequency of a rectangular cantilever is given as:

(8) $$f_{0}=\frac{1}{2\pi }\sqrt{\frac{E}{\rho }}\times \frac{t}{l^{2}}$$

This equation states that the resonant frequency of a rectangular beam is directly proportional to its thickness, and inversely proportional to its length. Therefore, the resonant frequency can be increased by either increasing the thickness and/or decreasing the length. A simplified form of above equation for a rectangular profile cantilever is given as:

(9) $$f_{0}=\frac{1}{\pi }\sqrt{\frac{k}{m}}$$

where *k* is the spring constant of the cantilever and *m* is its mass. The equation predicts the fundamental resonant frequency can be increased by either reducing the cantilever mass or increasing the cantilever spring constant. The cantilever mass can be reduced by changing its shape, size or profile. Since the spring constant of a cantilever is governed by its geometric properties at the fixed end, the changes should be done at the fixed end. Therefore, we reduced the area towards the fixed ends in Models #2, #3, #5 and #6. This reduction resulted in reduced spring constants for the models; and, as suggested by Equation 9, a reduction in their resonant frequencies. However, the change in area also reduced the mass of the models. Comparing the relative reduction in the spring constants and the masses, we observe the reduction is more pronounced in their spring constants, evident by a higher deflection exhibited by these models. Thus, we observe an increase in their sensitivities, expressed by their Δ*z* · *f*_0_ values (Table 3). The sensitivity values are evidently more inclined towards increasing the deflection.

From structural dynamics point of view, reduction in spring constant is undesirable, because it will decrease the resonant frequency of the cantilever. Therefore, another way to reduce the mass, while keeping the spring constant unchanged, is to change the cantilever profile. As mentioned above, geometric properties at the fixed end of the cantilever define its behaviour. Therefore, we may change the cantilever profile in a manner keeping the fixed-end thickness same and changing the thickness far from it. This scheme can be easily realized by trapezoidal profile cantilever (Model #4). It can be readily calculated that Model #4 has 25% less mass than #1 (Figure 2). Compared with the original rectangular profile (Model #1), the trapezoidal profile shows 57% increase in deflection, 9% increase in resonant frequency, and an overall 71% increase in the sensitivity (Table 3). The sensitivity can be further improved by modifying the shape of the trapezoidal cantilever (Models #5 and #6). By changing the shape, we observe 51% increase in deflection, 34% decrease in frequency and about 7% increase in sensitivity (Table 3). However if we compare Models #5 and #6 with the original Model #1, we observe about 138% increase in deflection, 25% decrease in frequency and 75% increase in sensitivity. Thus we see Models #5 and #6 have big advantages in producing deflections more than two times those of Model #1. Therefore, we may conclude that Models #5 and #6 are best suited for the sensing element of microcantilever biosensor. The modest decrease in their resonant frequencies can be compensated by employing differential readout techniques in measuring the deflections.

Thus far we discussed the deflection and vibration characteristics pertaining to the static mode of the microcantilevers. Static mode is used for determining the surface stress, diffusion or biomolecular recognition, whereas the dynamic mode is used as microbalance, thermogravimetry, or determining the amount of biomolecules adsorbed onto the cantilever. Dynamic mode uses the mass change induced resonant frequency change to calculate the amount of molecules adsorbed onto the functionalized surface of the microcantilever. Using dynamic mode, mass changes in the picogram range can be observed [31]. The fundamental resonant frequency is also critical when the microcantilevers are operated in the dynamic mode. In dynamic mode, the microcantilevers are excited to its fundamental resonant frequency. The adsorbed molecules increase the mass of the cantilever, and reduce the resonant frequency. The mass change and the frequency change are related as [31]:

(9) $$\Delta m=\frac{k}{4\pi^{2}}\left(\frac{1}{f_{1}^{2}}-\frac{1}{f_{0}^{2}}\right)$$

where Δ*m* is the mass added to the cantilever, and *f*_0_ and *f*_1_ are the fundamental resonant frequencies before and after the mass addition. It can be readily observed from Equation 9 that higher the fundamental resonant frequency higher will be the microcantilever sensitivity in measuring the mass change. Thus, we observe that for improving the microcantilever sensitivity, the high resonant frequency requirement for the static mode is also valid for the dynamic mode. Therefore, we can conclude that actuated whether in the static or dynamic mode, a high sensitive microcantilever should have high resonant frequency.

## 6. Conclusions

To improve the sensitivity of microcantilevers used in sensors, this study investigated rectangular and trapezoidal profile microcantilevers. For each profile, three cantilever designs were analyzed. The surface stress was successfully modelled by an in-plane tensile force applied to the top surface of the cantilevers. The finite element analysis investigation indicated that by changing the profile from rectangular to trapezoidal, the cantilever sensitivity is increased by 71%. Further, for each cantilever type, if we change only the shape, the sensitivity is increased by 17% for the rectangular and 7% for the trapezoidal cantilevers. However, if we combine the profile change with the shape change, the overall sensitivity is improved by 77%. Stress analysis showed that compared to the ultimate strength of silicon, the maximum stress induced in the models is negligible. We also showed that the high fundamental resonant frequency is a basic requirement for high sensitive cantilevers used in either static or dynamic mode. Based on the results of this investigation, we can conclude that the trapezoidal profile cantilevers has better deflection and resonant frequency characteristics than the rectangular, and hence can be efficiently used as the sensing element in microcantilever sensors.

## Figures and Tables

**Table 1.** Verification results comparing the experimental, analytical and simulation results.

**Surface Stress (N/m)**	**Max. Deflection (μm)**		
	**Exp.[1]**	**Analytical**	**Simulation**
0.05	0.89	1.11	1.14

**Table 2.** Comparison between analytical and simulation results for uniform width rectangular and trapezoidal cantilevers.

**Model**	**Max. Deflection (μm)**		**Frequency (kHz)**	
	**Analytical**	**Simulation**	**Analytical**	**Simulation**
#1	0.28	0.28	4.79	4.91
#4	0.43	0.44	5.59	5.33

**Table 3.** Comparison between simulation values for maximum deflection, fundamental resonant frequency, sensitivity and maximum induced stress.

**Model**	Δ***z*** (μm)	***f_0_*** (kHz)	Δ***z* · *f_0_***	***σ*_max_** (MPa)
#1	0.28	4.91	1.37	0.41
#2	0.53	3.04	1.61	1.13
#3	0.53	3.04	1.61	1.21
#4	0.44	5.35	2.35	0.79
#5	0.66	3.68	2.43	2.02
#6	0.67	3.68	2.46	1.99
Ref.	1.14	2.45	2.79	0.86
